# Cellulosic biofilm formation of *Komagataeibacter* in kombucha at oil-water interfaces

**DOI:** 10.1016/j.bioflm.2022.100071

**Published:** 2022-02-26

**Authors:** Guruprakash Subbiahdoss, Sarah Osmen, Erik Reimhult

**Affiliations:** Institute of Biologically Inspired Materials, Department of Nanobiotechnology, University of Natural Resources and Life Sciences (BOKU) Vienna, Austria

**Keywords:** Kombucha, Biofilm, *Komagataeibacteraceae*, Bacterial cellulose, Interfacial rheology, Oil-water interface, Interfacial tension, Scanning electron microscopy

## Abstract

Bacteria forming biofilms at oil-water interfaces have diverse metabolism, they use hydrocarbons as a carbon and energy source. Kombucha is a fermented drink obtained from a complex symbiotic culture of bacteria and yeast, where acetic acid bacteria present in kombucha use sugars as a carbon source to produce cellulosic biofilms. We hypothesize that *Komagataeibacteraceae* in kombucha can adsorb to and use hydrocarbons as the sole energy source to produce cellulosic biofilms. Hence we characterized a kombucha culture, studied bacterial adsorption and cellulosic biofilm formation of kombucha at the *n*-decane or mineral oil-kombucha suspension interface. The cellulosic biofilms were imaged using fluorescence microscopy and cryo-scanning electron microscopy, and their time-dependent rheology was measured.

*Komagataeibacter,* the dominant bacterial genus in the kombucha culture, produced cellulosic biofilms with reduced cellulose biomass yield at the oil-kombucha suspension interfaces compared to at the air-kombucha suspension interface. The presence of biosurfactants in the supernatant secreted by the kombucha microbes led to a larger and faster decrease in the interfacial tension on both oil types, leading to the formation of stable and elastic biofilm membranes. The difference in interfacial tension reduction was insignificant already after 2 h of biofilm formation at the mineral oil-kombucha suspension interface compared to kombucha microbes resuspended without biosurfactants but persisted for longer than 24 h in contact with *n*-decane. We also demonstrate that *Komagataeibacter* in kombucha can produce elastic cellulosic biofilms using hydrocarbons from the oil interface as the sole source of carbon and energy. Thus *Komagataeibacter* and kombucha shows the potential of this system for producing valued bacterial cellulose through remediation of hydrocarbon waste.

## 1 Introduction

Bacteria form biofilms at interfaces (solid-liquid, liquid-liquid, and liquid-air) that can impact healthcare, pharmaceuticals, food, and oil recovery industries. Mostly biofilms are detrimental, e.g., biofilm formation in drinking water pipelines, on industrial surfaces such as heat exchangers, and processing surfaces in food industries [[1], [2], [3], [4]]. However, biofilms can be beneficial in the areas of bioremediation [5,6] and wastewater treatment [[7], [8], [9]]. In bioremediation, bacterial biofilms form on liquid, e.g., oil-water interfaces. Bacteria forming biofilms at oil-water interfaces have diverse metabolisms, which allow them to use hydrocarbons, i.e., oils, including crude oil, as carbon and energy sources [10]. The product of bioremediation can also be useful, e.g., when the bacteria produce organic compounds or polymers of commercial value. One example of a biopolymer produced by bacteria in high demand is bacterial cellulose.

Hence, understanding how bacteria adsorb to and form biofilms at oil-water interfaces is essential and could have a tremendous societal and economic impact. However, the bacterial adsorption and biofilm formation at liquid interfaces differ significantly from the well-investigated process of biofilm formation on solid interfaces [11,12].

Recently, we studied the bacterial adsorption and biofilm formation of *P. aeruginosa*, *S. aureus*, and *S. epidermidis* at the *n*-decane-water interface, with the focus on understanding the relationship between bacterial physical characteristics, i.e., wettability and excretion of surfactants, and biofilm formation. Our results demonstrated that hydrophobic *P. aeruginosa* and hydrophilic *S. epidermidis* adsorbed rapidly to the interface and formed biofilms that are stable and elastic compared to hydrophilic *S. aureus* [13]. These findings suggest that bacterial adsorption and biofilm formation at oil-water interfaces depend both on the bacterial wettability and on biosurfactants secreted by the bacteria that modify the interface secreted by the bacteria [13]. Biosurfactants are amphiphiles and polymers secreted by the bacteria that are surface-active. Thereby they affect the bacteria's ability to adsorb to and remodel liquid interfaces. This can greatly influence the success and lifestyle of bacteria at oil-water interfaces important for their use in applications.

Kombucha, a traditional fermented tea drink, has recently gained significant popularity [[14], [15], [16]]. Kombucha tea is obtained from a complex culture of acetic acid bacteria (AAB; *Acetobacter*, *Gluconobacter,* and *Komagataeibacter*) [17], yeasts (*Saccharomyces cerevisiae*, *Brettanomyces bruxellensis*, *Schizosaccharomyces pombe,* and *Zygosaccharomyces rouxii*) [18] and often, but not always, lactic acid bacteria (LAB; *Lactobacillus*, *Lactococcus*) [19]. The symbiotic culture initiates the fermentation of tea and sugars, producing a floating biofilm over several weeks. At the beginning of the fermentation, the yeasts produce an invertase enzyme that hydrolyses sucrose into glucose and fructose, which bacteria and yeasts use as energy sources. The yeasts metabolize the sugars to produce ethanol. AAB in kombucha oxidise ethanol to produce acetic acid and gluconic acid, thereby lowering the overall pH of the fermenting kombucha. Further, the AAB need oxygen (obligate aerobes) for fermentation, use sugars as carbon and energy source to produce cellulosic biofilms or pellicle. The pellicle on the surface of the aqueous phase in kombucha increases the access to oxygen for the microbes embedded in the cellulose matrix [20]. In general, *Komagataeibacter* (historically known under several names, including *Gluconacetobacter* and *Acetobacter*) species possess the ability to produce cellulose and is the dominant bacterial genus in kombucha [[21], [22], [23], [24]].

We hypothesize that AAB in kombucha, specifically *Komagataeibacter,* can use hydrocarbons as the sole energy source to produce cellulose and thereby produce cellulosic films at oil-kombucha suspension interfaces without the addition of sugars. AAB in kombucha adsorbing to and forming stable cellulosic biofilms at the oil-water interface while using oil as their energy source could be developed to remediate hydrocarbon wastes into a valuable polymer resource. We characterized kombucha culture in terms of biochemical properties and bacterial composition, studied the adsorption and cellulosic biofilm formation of AAB from kombucha at aqueous interfaces with *n*-decane and mineral oil, respectively, to investigate our hypothesis. The bacterial adsorption and structure of the cellulosic biofilm formation at the interface were examined using fluorescence microscopy and cryo-scanning electron microscopy (SEM). A drop shape analyzer was used to measure the interfacial rheology of the cellulosic biofilm layer at the oil-kombucha suspension interface as a function of time.

## 2 Materials and methods

### 2.1 *Microorganism and culture conditions*

The starter culture, i.e., the kombucha pellicle, was kindly provided by our colleague Dr. Ronald Zirbs, BOKU, Vienna, Austria. To characterise AAB present in kombucha culture, we isolated and studied the morphology and Gram nature of the bacteria. The biochemical characterisation involves acetic acid fermentation and overoxidation of ethanol. Isolation of AAB was carried out on a glucose-ethanol medium (GEM: glucose, 15 g L^-1^; ethanol, 0.5%; peptone, 3 g L^-1^ and yeast extract, 3 g L^-1^; agar-agar, 15 g L^-1^) culture plate and incubated at 28 °C for 24 h. Further, the morphology and Gram nature of isolated bacteria were determined as described elsewhere [25]. The purified isolate was cultured on Carr medium (yeast extract, 30 g L^-1^; bromocresol green, 0.02 g L^-1^; agar-agar, 20 g L^-1^; and ethanol, 2%) and incubated at 28 °C for 5 days. The colour change in the Carr medium plates was observed and photographed every 24 h.

The bacterial diversity in kombucha was determined using a 16S Microbiome next generation sequencing (NGS) assay (ViennaLab Diagnostics GmbH, Vienna, Austria). Briefly, DNA was extracted using a DNA isolation kit (Norgen Biotek Corp, Ontario, Canada), a specific region of the 16S rRNA gene was amplified and sequenced. Then the generated sequence was identified using the NCBI 16S database (www.ncbi.nlm.nih.gov). The DNA extraction and sequencing of the bacteria were outsourced to Lab4more GmbH (Munich, Germany).

The standard culture medium used in the experiments was the HS medium described by Hestrin and Schramm [26]. HS liquid medium contained glucose (20 g L^-1^), peptone (5 g L^-1^), yeast extract (5 g L^-1^), and disodium phosphate (2.7 g L^-1^) in Milli-Q water (1 L). The inoculum culture was prepared by transferring a small piece of kombucha pellicle to 50 mL HS medium in a glass bottle, followed by static cultivation at 28 °C for 3 days. The cells were harvested by centrifugation at 5000 RPM for 5 min and diluted to OD_600 nm_ 0.05 in fresh HS medium or PBS. All materials used for medium preparation were purchased from Sigma-Aldrich.

### 2.2 *Bacterial adsorption and cellulosic biofilm growth at the oil-kombucha suspension interface*

90 mL of kombucha cell suspension (cells harvested from static culture and diluted to OD_600 nm_ 0.05 in fresh HS medium) was added to 10 mL of *n*-decane (>99% pure, Sigma-Aldrich) or mineral oil (Sigma-Aldrich) in a 250 mL glass bottle and vigorously shaken for 2 min to investigate the adsorption and cellulosic biofilm formation at the interface by the presence of bacteria. The resulting emulsion was statically incubated at 28 °C for up to 30 days. A suspension without oil phase (kombucha suspension-air interface) was used as control. To determine the presence of bacteria in the droplets and at interfaces, small volumes of emulsions obtained through the mixing of kombucha suspension with *n*-decane or mineral oil were pipetted onto a clean glass slide and stained using 3.34 mM SYTO 9 green fluorescent nucleic acid stain (Thermo Fischer Scientific) in PBS. The emulsions were stained using 25 μM Calcofluor White (Sigma-Aldrich) in PBS for 15 min to confirm the presence of cellulose at the oil-kombucha suspension interface. The images were obtained using 40x objective lens, fluorescence microscopy (Nikon Eclipse TE2000, Nikon Europe B.V., Austria) equipped with Nikon DS-U1 camera. The experiments were performed in biological triplicates, each with three technical replicates.

### 2.3 *Scanning electron microscopy (SEM) of cellulosic biofilms*

SEM was performed to examine the architecture of cellulosic biofilms at interfaces. The biofilms formed at the oil-kombucha suspension interface after 30 days were recovered and gently rinsed in PBS and fixed with 2.5% glutaraldehyde for 2 h at 4 °C followed by dehydration using ethanol series (35, 50, 70, 95, and 100%) incubated for 3 min in each. High-vacuum secondary electron imaging was performed using an Apreo VS SEM (Thermo Scientific, The Netherlands) at 500 V.

### 2.4 *Cryo-SEM of cellulosic biofilms*

High-pressure freezing, fracture, and cryogenic SEM imaging were performed to examine the inner structure of cellulosic biofilms formed at the various interfaces. The cellulosic biofilm samples were placed in specimen carriers and mounted on a sample holder. The samples were then frozen under high pressure using a high-pressure freezing system (Leica EM HPM100, Leica Microsystems Inc., Austria). This was followed by fracturing at -110 °C using a flat edge knife and sublimation at -95 °C for 5 min under the vacuum of 10^-7^ mbar in a freeze-fracture and etching machine (Leica EM ACE900). The samples were transferred to the SEM via a cryo-transfer system (Leica EM VCT500). High-vacuum secondary electron imaging was performed using an Apreo VS SEM at 500 V. During SEM imaging, the chamber temperature was maintained at -110 °C.

### 2.5 *Bacterial cellulose membrane*

After culturing, the cellulosic biofilms were washed with Milli-Q water and immersed in 0.1 M NaOH at 110 °C for 30 min to remove cells attached to bacterial cellulose membranes. Then the membranes were immersed in Milli-Q water for 3 days to warrant complete removal of biological substances, leaving the membrane at neutral pH. The purified cellulose membranes were lyophilised for 2 days. The membranes were weighed before and after lyophilisation. The masses are presented as hydrated and dry mass, respectively.

### 2.6 *Cellulosic biofilm rheology*

The rheological properties of the air-water interfaces were studied using the pendant drop method, *i.e*., Drop Shape Analyzer DSA30 (Krüss GmbH, Germany). The kombucha suspension was prepared from kombucha culture with supernatant, diluted to OD_600 nm_ 0.05 in fresh HS medium. A cuvette was filled with the prepared kombucha suspension. Then, an air bubble of 5 μL was formed and held at the tip of an inverted needle immersed in the kombucha suspension maintained at 28 °C. The air bubble size was kept constant, and the rheological properties were recorded as a function of time for 13 h.

The interfacial tensions of the *n*-decane or mineral oil-kombucha suspension interfaces were measured using a Drop Shape Analyzer DSA30 as described elsewhere [10,13]. A cuvette was filled with kombucha suspension of OD_600 nm_ 0.05 prepared in PBS. Then, droplets of 5 μL of *n*-decane or mineral oil were formed and held at the tip of an inverted needle immersed in the kombucha suspension maintained at 28 °C. The drop was aged in suspension for 24 h and until 7 days, and the rheological properties were measured. To determine the viability of bacteria and cellulose formation after 7 days, *n*-decane or mineral oil droplet was pipetted from the tip of the inverted needle onto a clean glass slide. Subsequently, the droplet was stained using vitality staining solution (3.34 mM SYTO 9 and 20 mM PI (Thermo Fischer Scientific)) and 25 μM Calcofluor White (Sigma-Aldrich) in PBS for 15 min. The images were obtained using a 40x objective lens by fluorescence microscopy on a Nikon Eclipse TE2000 (Nikon Europe B.V., Austria) equipped with a Nikon DS-U1 camera. The influence of kombucha microbes with supernatant and only PBS on the oil interface properties were also analysed.

The dynamic mechanical modulus of the oil-water interface during adsorption and cellulosic biofilm formation was measured using the Oscillation Drop Module (ODM) of the Drop Shape Analyzer DSA30. The oscillation of the oil drop at a volume percentage change of 20% and a frequency of 1 Hz was performed. From the time-dependent deformation of the drop shape, the elastic (storage) modulus (E′) and viscous (loss) modulus (E'′) were quantified every 24 h [27]. Large-scale deformations of the interface were investigated by manually removing ∼4.5 μL of oil from the 5 μL droplets and observing the deformation of the interface film before reinjecting the same liquid volume. The experiments were performed in biological triplicates.

### 2.7 *Statistics*

Data are presented as a mean with standard deviation. ANOVA tests were performed, followed by a Tukey's HSD post-hoc test, and a p-value <0.05 was considered significant.

## 3 Results

### 3.1 Gram-negative, rod-shaped AAB of *Komagataeibacter* genus dominantly present in Kombucha culture

Bright-field microscopy images of the Gram-stained isolates showed that the bacteria present in the Kombucha cellulosic biofilms were Gram-negative, short, and rod-shaped bacteria (Supplementary Fig. S1). Incubation of isolated bacteria on Carr medium showed a colour change from blue (colour of bromocresol green in Carr medium) to yellow due to acetic acid fermentation (Supplementary Fig. S2). Further, reversion of blue colour after 120 h of incubation indicates the overoxidation of acetic acids to CO_2_ (Supplementary Fig. S2). Based on the morphology, Gram nature, and biochemical characterisation, we confirm the presence of AAB in Kombucha culture.

16S Microbiome NGS assay showed that there are five different bacterial phyla in our kombucha culture, i.e. *Firmicutes*, *Cyanobacteria*, *Spirochaetes*, *Bacteroidetes* and *Proteobacteria*. Among these phyla, 98.8% of the sequences were assigned to the *Proteobacteria*, out of which 95.9% belong to *Komagataeibacter* genus of the *Acetobacteraceae* family. *K. sucrofermentans* (49.4%), *K. rhaeticus* (27.2%) and *K. hansenii* (18.8%) formed a total of 95.4% out of the identified species in *Komagataeibacter* genus (Supplementary Fig. S3). *Komagataeibacter* are well-known producers of cellulose and can be present in kombucha [21,23]. Hence, the strong cellulose production observed by our kombucha culture must be attributed to these dominant *Komagataeibacter* species.

### 3.2 *Komagataeibacter* in kombucha attached and grew on both *n*-decane and mineral oil emulsion droplets

**Fig. 1:**
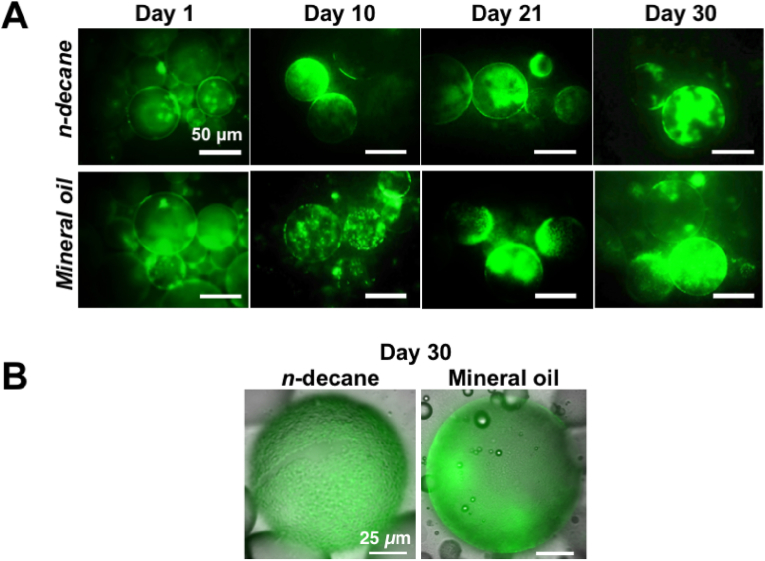
Bacteria adsorbed to and stabilized oil droplets for at least 30 days. **A)** Representative fluorescence microscope images of *Komagataeibacter* adhering to oil droplets after 1, 10, 21, and 30 days of incubation at 28 °C. The scale bars denote 50 μm. **B)** Representative superimposed bright-field and fluorescence images of *Komagataeibacter* adhering to oil droplet after 30 days of incubation. The scale bars denote 25 μm. The emulsions of *n*-decane or mineral oil in microbial dispersions obtained through mixing kombucha suspension with *n*-decane or mineral oil were stained using 3.34 mM SYTO 9 green fluorescent nucleic acid stain. (For interpretation of the references to colour in this figure legend, the reader is referred to the Web version of this article.)

Kombucha suspension and *n*-decane or mineral oil were mixed vigorously to form an emulsion, which was then allowed to settle and cultured at 28 °C to demonstrate the bacterial adsorption and cellulosic biofilm formation at the oil-kombucha suspension interface. The oil droplets in the sample containing bacteria were extracted, stained with 3.34 mM SYTO 9, and examined by fluorescence microscopy every 24 h (Fig. 1). The oil droplets of *n*-decane and mineral oil formed in the presence of *Komagataeibacter* in kombucha culture remained highly stable, which made the extraction and imaging possible (Fig. 1A). *Komagataeibacter* adhered to both types of oil droplets were visible and stabilized droplets for at least 30 days (Fig. 1B).

The presence of cellulose at the oil-kombucha suspension interface was confirmed by staining emulsions using 25 μM Calcofluor White (Sigma-Aldrich) and 3.34 mM SYTO 9 in PBS for 15 min. Blue colour surrounding the oil droplets indicates the cellulose formation on the oil droplets (Supplementary Fig. S4A). Calcofluor White also binds to chitin in the cell wall of yeasts (red arrows indicate yeasts in Supplementary Fig. S4A). The superimposed green and blue fluorescence image showed the *Komagataeibacter* and cellulose biofilm formed on the *n*-decane droplets (Supplementary Fig. S4B).

### 3.3 *Komagataeibacter* formed cellulosic biofilm pellicles at the *n*-decane or mineral oil-kombucha suspension interface

**Fig. 2:**
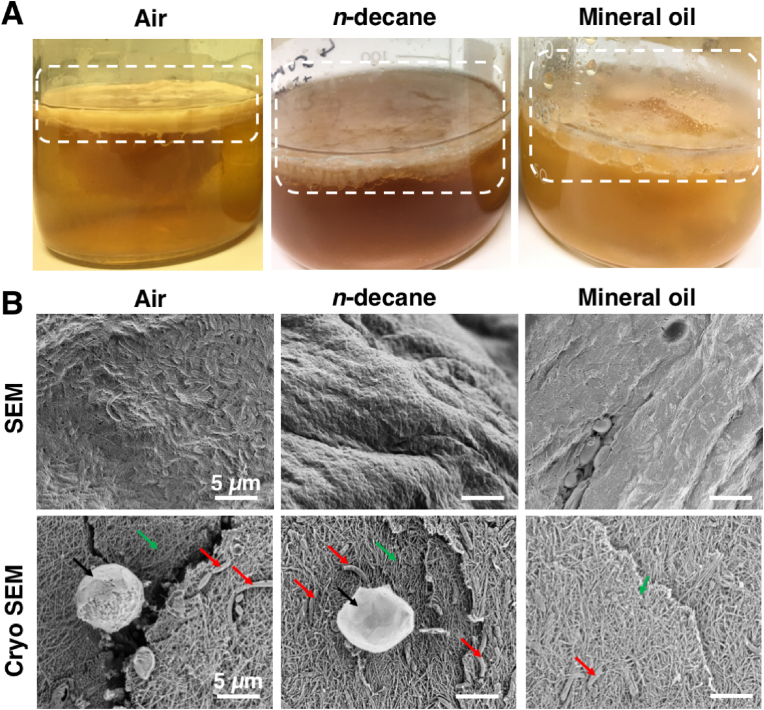
Cellulosic biofilm formation of kombucha culture at the kombucha suspension-air/oil interface. **A**) Representative photographs of cellulosic biofilms formed at the interface after 30 days. The regions within the dashed squares show the membrane pellicle formed at the interfaces. Bacteria produced a thick pellicle layer at the kombucha suspension-air interface compared to the kombucha suspension-oil interfaces. **B**) Scanning electron micrographs of the pellicle show the surface structure of an interconnected network of cellulose fibres secreted by the *Komagataeibacter*. The high-pressure-frozen, freeze-fractured pellicles imaged by cryo-scanning electron microscopy reveal the internal structure of the pellicle cellulose matrix embedding *Komagataeibacter* and yeast. Arrows in red point to *Komagataeibacter,* black arrows point to yeast*,* and green arrows point to the cellulose matrix. The scale bars denote 5 μm. (For interpretation of the references to colour in this figure legend, the reader is referred to the Web version of this article.)

The emulsions of *n*-decane or mineral oil in kombucha suspensions obtained from vigorous mixing were allowed to settle and incubate at 28 °C for 30 days. The oil and water phases separate, leaving an emulsion interface where the growth of cellulosic biofilms can be observed over many days (Fig. 2A). In controls without oil, i.e., leaving the kombucha suspension in contact with air, thick cellulosic biofilm pellicles were formed (Fig. 2A). This can be attributed to that *Komagataeibacter* are obligate aerobes, i.e., they need a good oxygen supply for growth [20]. While the oxygen permeability of alkanes is high, the oxygen supply will be more limited at the oil-water interface than at the cellulosic air-water interface.

Additionally, we imaged the pellicles recovered from the interfaces after 30 days using SEM to obtain their surface and internal structures. The surface of the pellicle revealed an interconnected network of cellulose fibres secreted by *Komagataeibacter* (Fig. 2B). High-pressure freezing followed by freeze-fracture and cryo-SEM of the pellicles showed their internal structure. An interconnected network of cellulose fibres (green arrows) embed *Komagataeibacter* (red arrows) and yeasts (black arrows) dispersed inside the pellicles (Fig. 2B). High-resolution scanning electron micrographs of the protoplasts of the yeasts, with the thick glucan fibrils typical of *Saccharomyces*, are shown in Supplementary Fig. S5.

The kombucha pellicles were harvested, all microbes were removed and weighed both in the hydrated and lyophilised states to compare the amount of cellulose produced at the various interfaces. Supplementary Fig. S6 shows photographs of the pellicles at different stages of purification. The mass of cellulose harvested at the oil-water interfaces was nearly one order of magnitude less than cellulose produced at the air-water interface (control, Table 1).

**Table 1:** Hydrated and dry masses of cellulose produced at the kombucha suspension-oil and kombucha suspension-air (control) interfaces after 30 days.

Conditions	Air	*n*-decane	Mineral oil
Hydrated mass (g/100 mL)	13.5 ± 1.2	6.30 ± 1.1 **	6.5 ± 2.3 **
Dry mass (g/100 mL)	1.2 ± 0.1	0.15 ± 0.10 **	0.16 ± 0.20 **

** denotes significance (p < 0.01) compared to the control.

### 3.4 Cellulosic biofilm formation at the air-kombucha suspension interface and its interfacial rheology

**Fig. 3:**
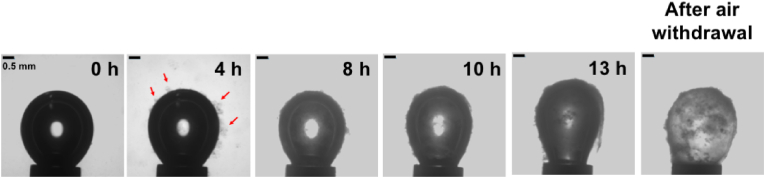
Cellulosic biofilm formation at the air-kombucha suspension interface as a function of time. An air bubble was formed at the tip of an inverted needle and aged in the kombucha suspension maintained at 28 °C for 13 h. The scale bars denote 0.5 mm. Red arrows indicate the formation of cellulose (fibre-like structures) starting to be visible at the interface after 4 h. (For interpretation of the references to colour in this figure legend, the reader is referred to the Web version of this article.)

Cellulosic biofilm formation at the air-kombucha suspension interface and its rheological properties were studied using the pendant drop method. The kombucha suspension was prepared from kombucha culture with supernatant diluted to OD_600 nm_ 0.05 in fresh HS medium. Briefly, an air bubble was formed at the tip of an inverted needle in kombucha suspension maintained at 28 °C. The air bubble size was kept constant, and the interfacial tension was recorded as a function of time for 13 h. Fig. 3 shows photographs of how a thick and inhomogeneous cellulosic biofilm forms at the air-kombucha suspension interface over time.

**Fig. 4:**
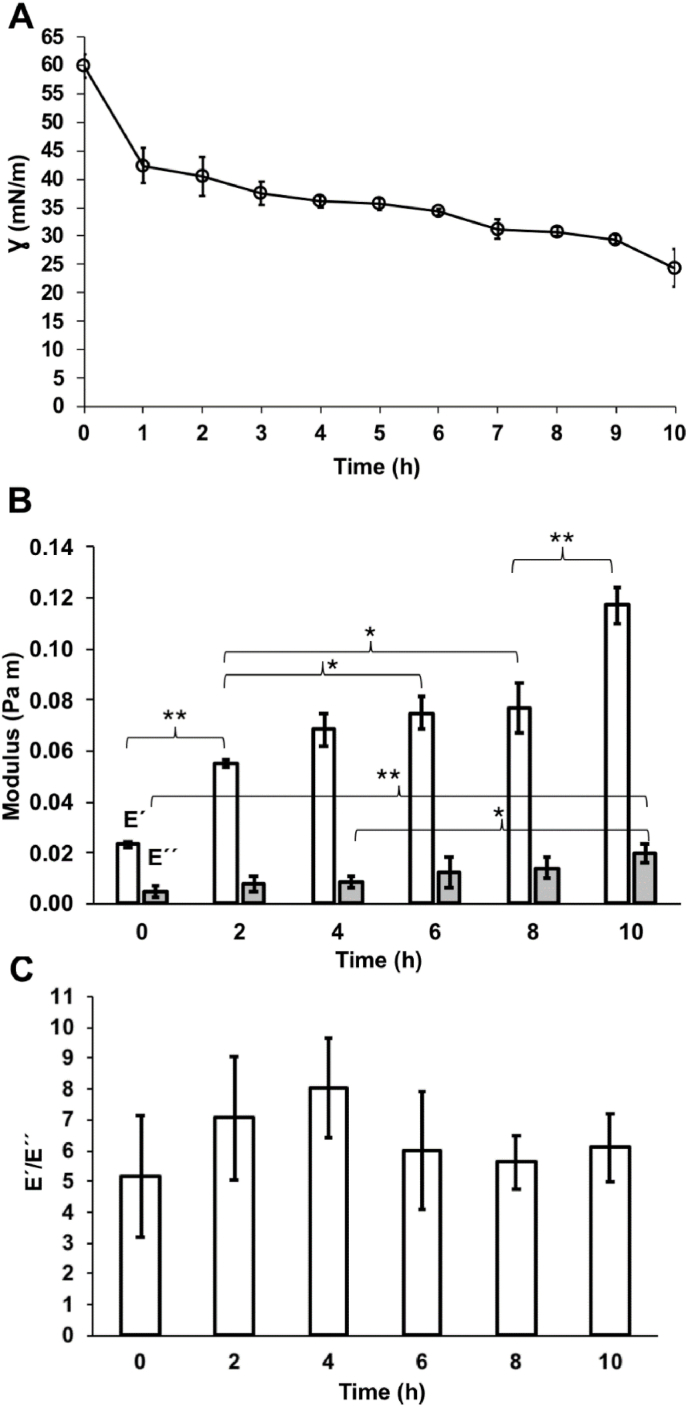
Rheology of bacterial adsorption and cellulosic biofilm formation at the air-kombucha suspension interface. **A**) Interfacial tension (γ.) as a function of time measured at the interface. **B**) Interfacial elasticity measurements (E′- elastic modulus and E'′- viscous modulus) of bacterial adsorption and cellulosic biofilm formation at the interface performed by the oscillating drop method. The E′ and E'′ were measured every 2 h. Error bars represent standard deviations over three replicates. * indicates a statistically significant difference of means with *p* < 0.05. ** indicates a statistically significant difference of means with *p* < 0.01. **C**) The ratio of elastic to viscous modulus (E'/E'′) of the interface as a function of time. Error bars represent standard deviations over three biological replicates. ANOVA tests were performed, and no significant differences were observed between the time points.

After 4 h, fibre-like structures (indicated by red arrows) are visible at the air-kombucha suspension interface, indicating the formation of cellulose. With increasing time, a thick membrane was gradually formed at the interface (Fig. 3). Bacterial adsorption and cellulosic biofilm formation at the air-kombucha suspension interface decreased the interfacial tension, with the fastest and largest drop in interfacial tension occurring within the first hour (Fig. 4A). During this time, the cellulose membrane has only started to form, indicating that bacteria or biosurfactant adsorption dominates over cellulose membrane formation for the reduction in interfacial tension. We modelled the shape deformation in response to the perturbation following Russev et al. [27]. to obtain the dynamic mechanical modulus (storage or elastic modulus, E′, and loss or viscous modulus E'′) of the interface. The oscillation of the air bubble had an amplitude of 20% of its volume. It was applied at a frequency of 1 Hz for 10 s every 2 h of the bacterial adsorption and biofilm formation. Fig. 4B shows the change in the dynamic modulus of the interface. Both elastic and viscous moduli increased over time. However, the loss modulus remained lower than the elastic modulus at all time points, indicating that these adsorbed layers are predominantly elastic (Fig. 4B). The interface reached its highest ratio of elastic to loss modulus (E'/E'′>8) at 4 h, but the ratio gradually reduced to E'/E'′≈6 after 6 h (Fig. 4C), where it remained. When the air bubble was removed after 13 h of biofilm formation, the cellulosic membrane formed at the interface did not deform. It remained stably attached to the inverted needle tip (Fig. 3, Supplementary Video S1).

Supplementary data related to this article can be found at https://doi.org/10.1016/j.bioflm.2022.100071.

The following is the supplementary data related to this article:

Multimedia component 1 Multimedia component 1

### 3.5 *Komagataeibacter* adsorption and cellulosic biofilm formation decreased the interfacial tension and increased the elasticity of the oil-kombucha suspension interface over time

**Fig. 5:**
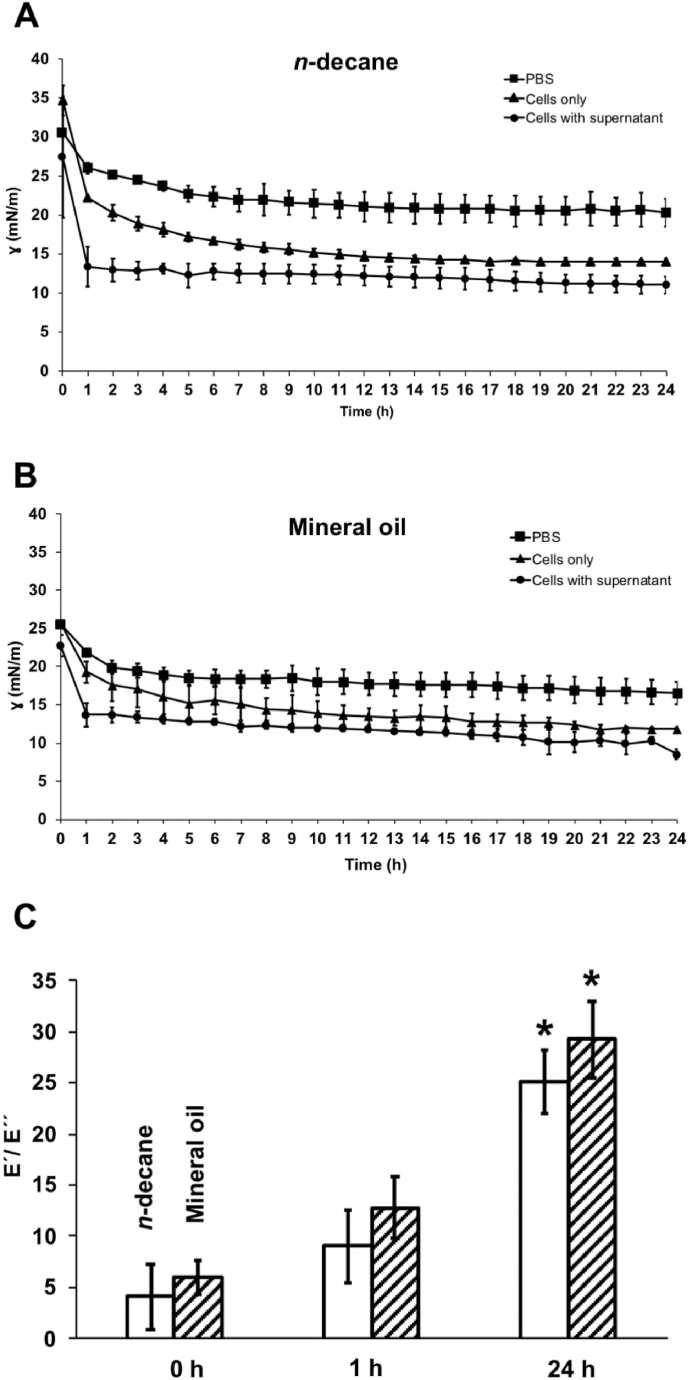
Interfacial tension (γ) as a function of time measured at the interface between **A**) *n*-decane and kombucha suspension, and **B**) mineral oil and kombucha suspension, in PBS or supernatant and only PBS as a control. **C**) The ratio of elastic to viscous modulus (E'/E'′) of the interface between oil and kombucha suspension with supernatant at 0 h, 1 h, and 24 h. Error bars represent standard deviations over three replicates. ANOVA tests were performed. * indicates significance (*p* < 0.05) compared to 0 h and 1 h for their corresponding oil types. No difference in E'/E'′ was observed between oil types.

Pendant drop measurements were performed on droplets of *n*-decane or mineral oil held at the tip of an inverted needle in kombucha suspension (OD_600 nm_ 0.05). In addition to the complete kombucha suspension, we performed measurements on resuspended kombucha microbes after removing biosurfactants present in the supernatant by centrifuging the kombucha suspension at 5000 RPM for 5 min, washing with PBS, and diluting to OD_600 nm_ 0.05 in PBS. The oil droplet size was kept constant, and the interfacial tension was recorded as a function of time for 24 h (Fig. 5A and Fig. 5B). We measured PBS against *n*-decane or mineral oil as a control.

The decrease in interfacial tension observed in the control measurements can be attributed to low-Mw alkane and aromatic impurities present in the as-purchased oils. The decrease in interfacial tension caused by bacterial adsorption to the interface was, in all cases, faster and larger than in the control experiments. The largest decrease in interfacial tension was observed for *n*-decane. Biosurfactants in the supernatant of the bacteria suspension led to a larger and faster interfacial tension decrease for both oil-water interfaces compared to kombucha resuspended in PBS without biosurfactants. However, the final value of the interfacial tension was significantly (p < 0.05) lower with mineral oil compared to *n*-decane. In the experiments with both oil types, the presence of biosurfactants in the supernatant led to a larger and faster decrease in the interfacial tension. The presence of biosurfactants reduced the interfacial tension to *n*-decane significantly (p < 0.05) compared to kombucha resuspended in PBS without biosurfactants in contact with *n*-decane. However, in contact with mineral oil, the influence of biosurfactants was significant (p < 0.05) only up to 2 h. Thereafter, no significant difference in the interfacial tension was observed compared to kombucha resuspended in PBS (Fig. 5B). In addition, we analysed the influence of adding pure supernatant. The microbes-free supernatant was prepared before the experiments by centrifugation of kombucha culture at 5000 rpm for 5 min. After centrifugation, the supernatant was filtered through a 0.22 μm filter. The decrease in interfacial tension was similar to the measurements on microbes with supernatant in both the oil types (Supplementary Fig. S7).

The shape deformation of the drop is analysed as described above at time points 0 h, 1 h, and 24 h of the bacterial adsorption and cellulosic biofilm formation. The E'′ remained significantly lower compared to E′ at all time points indicating the adsorbed layer remained elastic (Supplementary Fig. S8). At 1 h, a significant increase in elastic (E' = 0.04 Pa m) and viscous (E'′ = 0.004 Pa m) moduli of the droplet interface was observed on *n*-decane, yielding a dynamic elasticity to viscosity ratio of ∼9 (Fig. 5C). A similar significant increase in elastic (E' = 0.04 Pa m) and viscous (E'′ = 0.003 Pa m) moduli of the mineral oil droplet interface was observed (Supplementary Fig. S8). At 24 h, the elasticity of the oil droplet interface reached its highest (E'/E'′>25) for both oil types with no significant differences observed between the oil types (Fig. 5C).

### 3.6 Cellulosic biofilms displayed reversible buckling upon interfacial area reduction

**Fig. 6:**
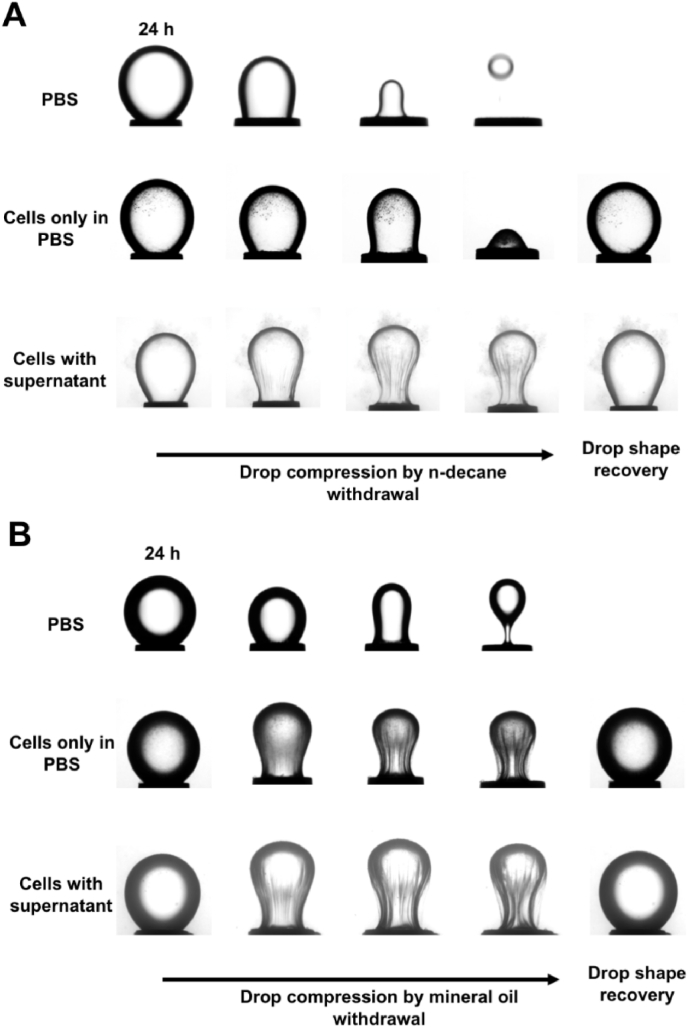
Deformation of interfaces of kombucha suspensions (PBS or microbes with supernatant) in contact with **A**) *n*-decane or **B**) mineral oil droplet in response to a large volume reduction. A droplet of oil (5 μL) was formed at the tip of an inverted needle and aged in the kombucha suspension for 24 h. The aged drop is compressed manually by withdrawing 90% of the oil droplet volume and then reinjecting it after observing the compression of the interface. The complete process (withdrawal and reinjection of oil droplet volume) took ∼1 min. Supplementary Videos (Videos S2–S7) were recorded, and snapshots at different stages are displayed in the figure.

Biofilms at liquid interfaces are membranes held together by an extracellular matrix that could respond to large deformations by buckling. Therefore, we recorded videos of the deformation of the droplet interface after 24 h incubation with kombucha suspension in PBS or with supernatant and only PBS as control. The oil drop was compressed by removal of 90% of the volume (up to ∼4.5 μl of the ∼5 μl removed) of the *n*-decane or mineral oil droplet (Supplementary Videos S2–S7). A different behaviour was observed with the different oil types and kombucha suspensions (with and without biosurfactants). When the *n*-decane or mineral oil drop was compressed after 24 h incubation with kombucha suspension with supernatant (with biosurfactants), the oil-kombucha suspension interface formed as a stalk due to the reduction of the diameter of the droplet in contact with the syringe; this was followed by buckling for both *n*-decane and mineral oil droplets. These observations indicate a stable elastic biofilm formed at the interface (Fig. 6A and Fig. 6B, Supplementary Videos S4 and S7).

In the case without biosurfactants, when the *n*-decane drop was compressed after 24 h incubation with kombucha suspension resuspended in PBS, the biofilm layer formed at the interface was not an elastic membrane; it appeared unstable as the film desorbed (Fig. 6A, Supplementary Video S3). In contrast, a stable elastic biofilm was formed at the mineral oil-water interface when the aged oil drop was compressed manually by withdrawing 90% of the mineral oil droplet volume (Fig. 6B, Supplementary Video S6). When the volume of *n*-decane or mineral oil was reintroduced after compression, the droplet shapes recovered. The repetition of volume withdrawal and reintroduction of oil showed similar elastic behaviour as described above (Supplementary Videos S4, S6 and S7).

Supplementary data related to this article can be found at https://doi.org/10.1016/j.bioflm.2022.100071.

The following is the supplementary data related to this article:

Multimedia component 3 Multimedia component 3

Higher interfacial tension and a lower elastic to viscous moduli ratio seem to correlate with a globally non-elastic biofilm for kombucha resuspended in PBS. However, the differences between the samples are minor. We emphasize that the interfacial tension is a measure of the interface and not of the biofilm. It is expected to change with the adsorption of bacteria and surfactants but is less sensitive to the bulk of the biofilm, while the drop contraction tests probe the elasticity of the bulk biofilm.

### 3.7 *Komagataeibacter* restructured the oil-water interface to an elastic film for up to 7 days in the absence of growth medium

**Fig. 7:**
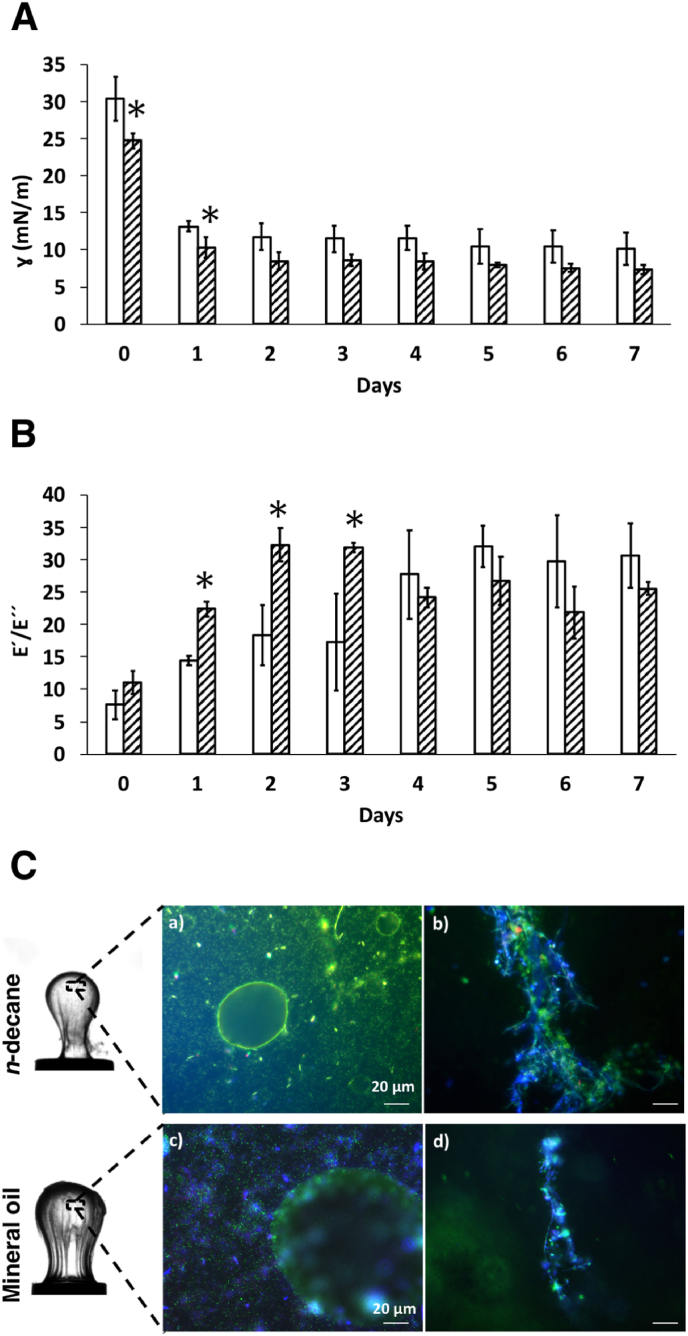
Interfacial rheology of cellulosic biofilms at the oil-water interface. **A**) Interfacial tension (γ) as a function of the number of days measured at the interface between i) *n*-decane (open box) and kombucha suspension, and ii) mineral oil (shaded box) and kombucha suspension. **B**) The ratio of elastic to viscous modulus (E'/E'′) of the interface between oil (i) *n*-decane (open box) and ii) mineral oil (shaded box)) and kombucha suspension at every 24 h for 7 days. The kombucha suspension was prepared by centrifugation of cells at 5000 RPM for 5 min and diluted to OD_600 nm_ 0.05 in PBS. * denotes significance (*p* < 0.05) compared to *n*-decane and kombucha suspension. **C**) Representative fluorescence microscope images of cellulosic biofilms formed at *n*-decane-kombucha suspension interface (a, b), and mineral oil-kombucha suspension interface (c, d) after 7 days. The scale bars denote 20 μm. The oil droplet was pipetted from the tip of the inverted needle onto a clean glass slide, stained using vitality staining solution (3.34 mM SYTO 9 and 20 mM PI) and 25 μM Calcofluor White in PBS for 15 min. Green colour indicates live cells stained with SYTO 9, red colour indicates dead cells stained with PI and blue colour represents cellulose stained with Calcofluor White. (For interpretation of the references to colour in this figure legend, the reader is referred to the Web version of this article.)

We performed pendant drop measurements on droplets of *n*-decane or mineral oil held at the tip of an inverted needle in a suspension of kombucha in PBS to demonstrate the use of hydrocarbons as a sole energy source for *Komagataeibacter*. The kombucha microbes were centrifuged at 5000 RPM for 5 min, washed with PBS, and diluted to OD_600 nm_ 0.05 in PBS to remove all potential sources of energy from the growth medium of the original suspension. The oil droplet size was kept constant, the interfacial tension and elasticity were recorded as described above, every 24 h for 7 days (Fig. 7).

A decrease in interfacial tension caused by bacterial adsorption was observed in the presence of either *n*-decane or mineral oil, which was evident already after one day. We did not observe any significant change in interfacial tension after day 1 for up to one week. A significant difference in interfacial tension between mineral oil and *n*-decane was observed at days 0 and 1, but we did not observe a significant difference between the oil types thereafter (Fig. 7A).

Both E′ and E'′ increased when the sinusoidal perturbation to the droplet volume was applied every 24 h of the bacterial adsorption and cellulosic biofilm formation. Again, the loss modulus, E'′, remained lower than the storage modulus, E′, at all times. The ratio of the storage to loss modulus increased with time and reached its highest (E'/E'′>32 at day 2 for mineral oil and on day 5 for *n*-decane). A significantly higher elastic to viscous moduli ratio was observed from day 1 to day 3 for mineral oil compared to *n*-decane (Fig. 7B).

When the drop was compressed manually by withdrawing 90% of its volume, the cellulosic biofilms formed at the oil-water interface after seven days of incubation showed the formation of a stalk and buckling for both *n*-decane and mineral oil droplets (Supplementary Fig. S9). This observation indicates that a stable elastic biofilm had formed at the interface. However, withdrawing the *n*-decane droplet on day 1 only reduced the volume while fully relaxing the interface without buckling (Supplementary Fig. S9A). The evolution of an increasingly elastic cellulosic biofilm over the entire 7-day period can be observed in Supplementary Fig. S9A. A comparison with Supplementary Fig. S9B shows that a stable and elastic biofilm formed faster at the mineral oil-kombucha suspension interface (within less than one day) than at the *n*-decane-kombucha suspension interface.

The viability of bacteria and production of cellulose after 7 days was verified using a live-dead fluorescence assay. A mineral oil or *n*-decane droplet was pipetted onto a clean glass slide, stained using vitality staining solution (3.34 mM SYTO 9 and 20 mM PI (Thermo Fischer Scientific)) and 25 μM Calcofluor White (Sigma-Aldrich) in PBS. The fluorescence microscopy images in Fig. 7C and Supplementary Fig. S10 show that *Komagataeibacter* at the *n*-decane/mineral oil-kombucha suspension interface after 7 days were alive and produced cellulose fibres (stained blue by the Calcofluor White dye).

## 4 Discussion

Adsorption of bacteria to a liquid interface is the first important step in biofilm formation. Biofilm formation at oil-water interfaces depends not only on bacterial surface properties and secreted biosurfactants that control adsorption to the interface but also on the bacterial ability to thrive and metabolize the hydrocarbon phase, i.e., use oil as a carbon and energy source [10].

During the kombucha fermentation process, yeasts hydrolyse sucrose into glucose and fructose by invertase. The yeasts metabolize the sugars to produce ethanol. AAB oxidises ethanol to acetic acid and finally to CO_2_ and water. The ability to oxidise acetic acid to CO_2_ is the major difference between genera *Acetobacter* and *Gluconobacter* [28]. The biochemical characterisation using Carr medium showed the ability of the isolated bacteria to oxidise ethanol to acetic acid through the colour change from blue to yellow and reversing to blue after 120 h of incubation, indicating the ability to oxidise acetic acid to CO_2_. This confirms that the isolated strain in the kombucha culture belongs to AAB. The 16S Microbiome NGS assay identified that the dominant AAB genus present in Kombucha is *Komagataeibacter*. Three species *K. sucrofermentans* (49.4%), *K. rhaeticus* (27.2%) and *K. hansenii* (18.8%) formed a total of 95.4% in *Komagataeibacter* genus and can, therefore, be expected to dominate the physicochemical behavior of the culture completely. AAB use glucose as the carbon source to produce cellulose at the air-water interface of the drink. Our results demonstrate that *Komagataeibacter* in kombucha culture produce cellulosic biofilms also at hydrocarbon oil-water interfaces. Further, that *Komagataeibacter* from this culture can utilise the hydrocarbon from the oil phase as its carbon and energy source when no sugar is added. We observe cellulose synthesis by viable bacteria for at least a week of culture at the oil-water interface in the absence of sugars. Hence, we are not observing the residual metabolism of the *Komagataeibacter.* The near absence of yeast in the biofilm at the oil water interface and the low solubility of alkanes in water supports that the substitute hydrocarbon metabolism is driven by the *Komagataeibacter* in contact with the oil phase. The biofilm properties, growth rates, and cellulose biomass production rate vary with the interface and conditions.

We observed different adsorption behaviours for different oil types, i.e., *n*-decane and mineral oil, by interfacial tension measurements using the pendant drop technique. The decrease in interfacial tension due to adsorption was observed for both oil types for microbes resuspended in PBS and native culture. However, the largest reduction of interfacial tension was observed for *n*-decane. A lowering of interfacial tension in response to the adsorption of bacteria has been reported in several studies [13,29,30]. This observation is expected from the Pickering-Ramsden effect [31,32] of reducing the interface area through the adsorption of the bacteria. Still, we observe that the presence of kombucha supernatant significantly contributed to the reduced interfacial tension of the *n*-decane-kombucha suspension interface. This finding aligns with our previous work on, *e.g*., *S. epidermidis,* showing that excreted biosurfactants can be instrumental to adsorption and biofilm formation at *n*-decane-water interfaces [13]. The contribution of the biosurfactants in the supernatant to the lowering of the interfacial tension was strongest during the initial adsorption. The difference between the native kombucha culture and supernatant-free resuspensions in PBS reduces over time. Presumably, this occurs because new biosurfactants and a cellulosic biofilm are synthesized and deposited at the interface over time also for the resuspended sample (Fig. 5A and B). It is striking that the difference was close to negligible already after a few hours for biofilm formation at the mineral oil-kombucha suspension interface (Fig. 5B) but persisted for a much longer time for the *n*-decane-kombucha suspension interface. This might correlate with the lower interfacial tension that further decreased with time of the mineral oil-kombucha suspension compared to the *n*-decane-kombucha suspension interface.

Furthermore, our results demonstrate that *Komagataeibacter* adsorption to oil-water interfaces lead to mechanically stable and elastic biofilms. *Komagataeibacter* from kombucha culture adsorbed rapidly to the interface and formed stable and elastic cellulosic biofilms to both *n*-decane and mineral oil-kombucha suspension interfaces within 24 h. Clearly, the rapid adsorption aided by the lowering of the interfacial tension by biosurfactants in the culture was instrumental to the fast synthesis of a mechanically robust biofilm.

When kombucha microbes were resuspended in PBS, *Komagataeibacter* needed more than 24 h to form stable elastic biofilms on the *n*-decane-kombucha suspension interface, to which the adsorption and biofilm formation at were slower compared to the mineral oil-kombucha suspension interface or in the presence of biosurfactants. This was demonstrated by the lack of elastic membrane properties when the interface was subject to large deformations, *e.g*., by a large reduction of interface area (Supplementary Fig. S9A). In analogy to our previous observations for the poor biofilm former *S. aureus* on *n*-decane-water interfaces, large perturbations of the oil-water interface displace the adsorbed bacteria from the interface when an interconnected extracellular matrix is not synthesized; the interface can relax as a liquid instead of an elastic membrane. However, with time all interfaces investigated in this study produced elastic membranes. The storage to loss modulus ratio reached its highest value on day 5 for the *n*-decane-kombucha suspension interface (Fig. 7B). From these results, it also seems plausible that in addition to adsorbing to and transforming the *n*-decane interface more slowly than the mineral oil interface, the *Komagataeibacter* could metabolize the diverse light hydrocarbons in mineral oil better than pure *n*-decane.

In addition to the lower and continuously decreasing interfacial tension between mineral oil and water, differences in the chemical composition likely played a major role in the faster synthesis of an elastic, cellulosic biofilm on mineral oil-kombucha suspension compared to *n*-decane-kombucha suspension interfaces. Decane (C_10_H_22_) consists entirely of hydrogen atoms and saturated carbon atoms. Only a small amount of other hydrocarbon impurities are present. In comparison, mineral oil (C_16_H_10_N_2_Na_2_O_7_S_2_) consists mostly of hydrocarbons but it also contains a smaller amount of sulfur, oxygen, and nitrogen compounds [33]. Nitrogen and oxygen already present in mineral oil could provide a head start for the bacteria to produce cellulosic biofilms. In kombucha culture, AAB uses nitrogen to produce acetic acid and gluconic acid and build the pellicle [17]. Similarly, other studies demonstrated the role of different carbon and nitrogen sources on cellulose production by *A. xylinum* [26,34].

AAB in kombucha are obligate aerobes, i.e., they require oxygen to function. In kombucha cultures, floating pellicle or cellulosic biofilm are formed at the air-water interface, supposedly to increase the access of oxygen and nitrogen to microbes embedded within the cellulose matrix as air contains oxygen (21%) and nitrogen (78%) as major components. We showed that *Komagataeibacter*, the predominant AAB genus in kombucha culture, adsorbed and produced cellulosic biofilms at the oil-kombucha suspension interfaces. Studies have demonstrated that a good supply of oxygen enhances bacterial cellulose production [35,36]. Chao et al. demonstrated that supply of oxygen-enriched air instead of air increased the bacterial cellulose production rate; the bacterial cellulose yield was enhanced from 11% in air to 18% in oxygen-enriched air [36]. A low supply of oxygen and nitrogen, therefore, can reduce the cellulose production rate and thereby affect the mechanical properties of cellulosic biofilms. As expected from these considerations, we found that the elastic (E' = 0.12 Pa m) and viscous (E'′ = 0.02 Pa m) moduli of cellulosic biofilms formed at the air-kombucha suspension interface in 10 h were significantly higher than the moduli (E' ≈ 0.03 Pa m, E'′ ≈ 0.001 Pa m) of kombucha biofilms formed at oil-kombucha suspension interfaces in 24 h. The cellulosic biofilms formed at the air-kombucha suspension interface are less elastic (E'/E'′≈ 6) compared to biofilms formed at oil-kombucha suspension interfaces (E'/E'′≈ 30). This was also confirmed from the deformation of the air bubble or oil droplet interface after 24 h incubation with kombucha suspension with supernatant. We tentatively attribute the reduced production and different mechanical properties of cellulosic biofilms formed at the oil-kombucha suspension interface compared to the air-kombucha suspension interface to the lower supply of oxygen at the oil-kombucha suspension interfaces.

Fischer and co-workers used interfacial shear rheology to determine kombucha biofilm growth at the air-water interface and its mechanical properties measured *in situ* for a duration of 7 days [37]. They reported that kombucha biofilm formation is a two-step process: i) an initial increase in both interfacial storage and loss modulus due to the adsorption of bacteria and surface-active proteins followed by a plateau and ii) a secondary increase in interfacial shear moduli due to bacterial cellulose excretion only after two days by *K. xylinus* in kombucha [37]. In our study, the mechanical properties of kombucha cellulosic biofilms formed at the air-water were measured using pendant drop experiment for a duration of 13 h. Similar to the above study, we observed an increase in both interfacial storage and loss moduli due to the adsorption of *Komagataeibacter* and biosurfactants (Fig. 4B). In contrast, we observed cellulosic fibre formed already at 4 h at the air-kombucha suspension interface (Fig. 3). However, the differences in the results could be attributed to the differences in the experimental setup.

The cellulosic biofilm membrane formed at the air-water interface did not deform and remained rigid and stably attached to the tip of the inverted needle (Fig. 3). Whereas, the cellulosic biofilm membranes formed at the oil-water interface showed the formation of a stalk and buckling for both *n*-decane and mineral oil droplets, indicating that a stable biofilm had formed at the interface but with a lower elastic modulus than at the air-kombucha suspension interface (Fig. 6). We also demonstrated that the cellulose production was significantly weaker when the oils were used as the energy source than for traditional kombucha culture that include sugars. However, the cellulose yield was significantly lower even for kombucha biofilms formed at oil-kombucha suspension interfaces in a glucose-containing medium than at the air-water interface (Table 1). The observed lower cellulose yield at the oil-kombucha suspension interfaces could at least in part be due to the low supply of oxygen and nitrogen compared to at the air-kombucha suspension interface.

The cooperative interactions between bacteria and yeast play an important role in the fermentation process of kombucha. Yeasts hydrolyse sucrose into glucose and fructose by invertase and produce ethanol via glycolysis*. Komagataeibacter* metabolize ethanol and use sugars to produce cellulosic biofilms at the air-water interface [20]. In the oil-kombucha suspension interface experiments, sugars were not added to the suspension. Therefore, the symbiotic role of yeasts in kombucha culture to hydrolyse sucrose to supply *Komagataeibacter* with energy was not active. However, the possibility that yeasts utilise hydrocarbon from the oil phase as its carbon and energy source cannot be ruled out, although it is unlikely to have been a major factor in our study. Several studies have reported the ability of *S. cerevisiae* to degrade *n*-alkanes [[38], [39], [40]]. However, yeast was not abundantly found at the interface. The poor solubility of alkanes in the aqueous phase makes this route an unlikely metabolic pathway for the cellulose synthesis observed at the oil-water interface of sugar-depleted suspensions.

The production of cellulosic biofilm membranes is influenced by different factors, such as strain type, culture medium, type of sugars, nutrient factors (carbon and nitrogen sources), pH, dissolved oxygen, temperature [[41], [42], [43]]. The medium used for kombucha culture in this study is Hestrin-Schramm, a widely used medium characterised by the presence of glucose as the only carbon source [26]. However, there are various studies focused on the evaluation of other carbon sources such as maltose, fructose, mannitol, molasses, and saccharose in order to increase the production yield of cellulose [43,44]. In one study, Marín et al. characterised a new *Xanthobacteraceae* strain that produced cellulosic biofilms when grown on naphthalene crystals as a carbon source [45]. We demonstrated that *Komagataeibacter* in kombucha could also use hydrocarbons as a source of carbon and energy to produced cellulosic biofilms. In the pendant drop measurements, *Komagataeibacter* in kombucha used oil as a carbon source, survived, and restructured the oil-kombucha suspension interface into an elastic cellulosic film for 7 days in the absence of other energy sources.

In conclusion, *Komagataeibacter*, the most dominant bacterial genus in kombucha culture, adsorbed and produced cellulosic biofilms at the oil-kombucha suspension interfaces. Using pendant drop measurements, we also demonstrated that *Komagataeibacter* in kombucha could adsorb to and modify the oil-kombucha suspension interface and promote cellulosic biofilm formation using hydrocarbons as the sole carbon and energy source. All interfacial cellulose films were elastic and grew more elastic over time, while the oil-kombucha suspension interface films showed a slower strengthening of mechanical integrity over time than air-kombucha suspension interface films. These findings could lead to future exploration of *Komagataeibacter* or kombucha culture in waste remediation, where it could convert hydrocarbons into a valuable biopolymer.

## Financial disclosure statement

This work was funded by the 10.13039/501100006380University of Natural Resources and Life Sciences, Vienna (BOKU Wien), Austria. The funders had no role in style design, data collection, analysis, decision to publish, or preparation of the manuscript.

## CRediT authorship contribution statement

**Guruprakash Subbiahdoss:** Study design, Conceptualization, Experiments, Formal analysis, Writing – original draft. **Sarah Osmen:** Experiments, Formal analysis, Writing – original draft. **Erik Reimhult:** Study design, Conceptualization, Formal analysis, Writing – original draft.

## Declaration of competing interest

The authors declare that they have no known competing financial interests or personal relationships that could have appeared to influence the work reported in this paper.

There are no conflicts of interest to declare.
