# Supplementary material for: Cellulosic biofilm formation of Komagataeibacter in kombucha at oil-water interfaces
Source: Biofilm. 2022 Feb 26;4:100071. doi: 10.1016/j.bioflm.2022.100071 (PMC8904243; doi:10.1016/j.bioflm.2022.100071)
Supplement: Multimedia component 4 [file mmc4.docx]

**Supplementary Materials**

**Cellulosic biofilm formation of *Komagataeibacter* in kombucha at oil-water interfaces**


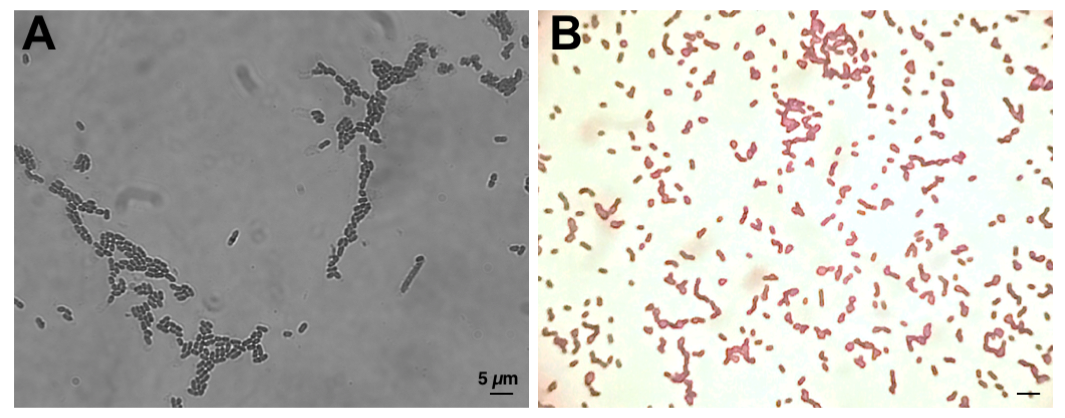


**Figure S1**. **A**) Bright-field micrograph and B) Gram-staining of isolated AAB from Kombucha suspension after 24 h incubation at 28°C on GEM plates. Bacteria stained pink, confirming Gram-negative bacteria.


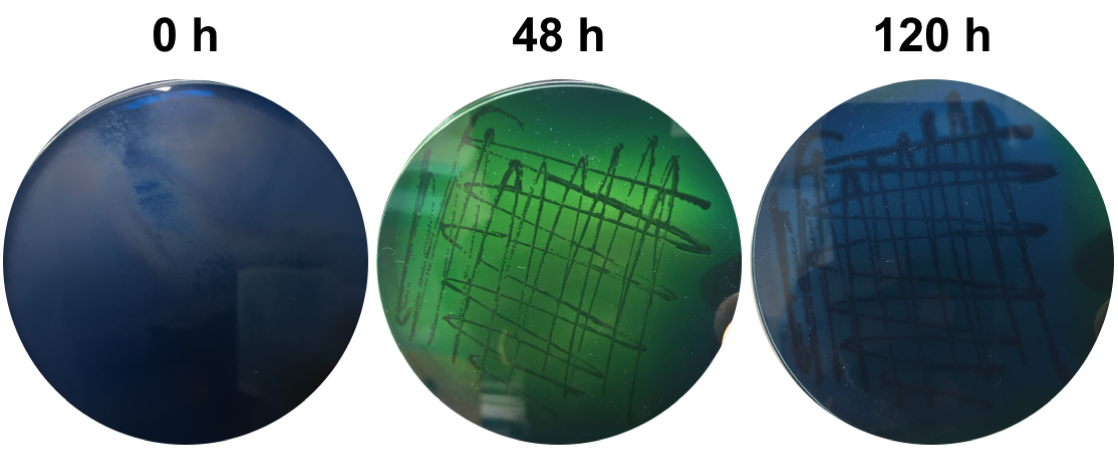


**Figure S2**. Colour change of Carr media from blue (0 h) to yellow (48 h) due to acetic acid fermentation and reversion of blue colour (120 h) due to overoxidation of acetic acids to CO_2_ by AAB.


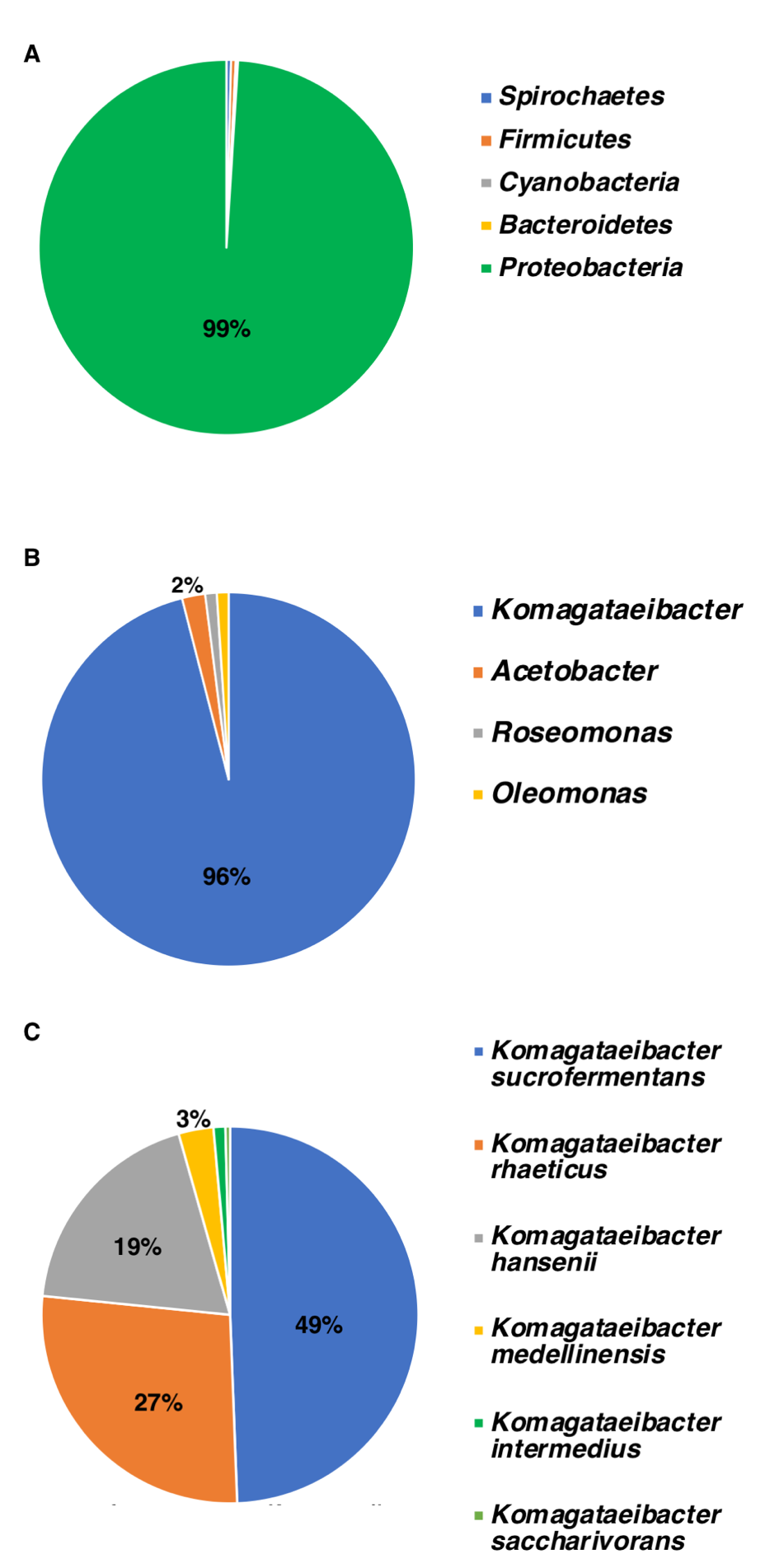


**Figure S3.** Taxonomical analysis of kombucha culture. The relative percentage of A) different bacterial phyla in kombucha culture, B) different genera in *Proteobacteria* phylum, and C) bacterial species in *Komagataeibacter* genus are shown.


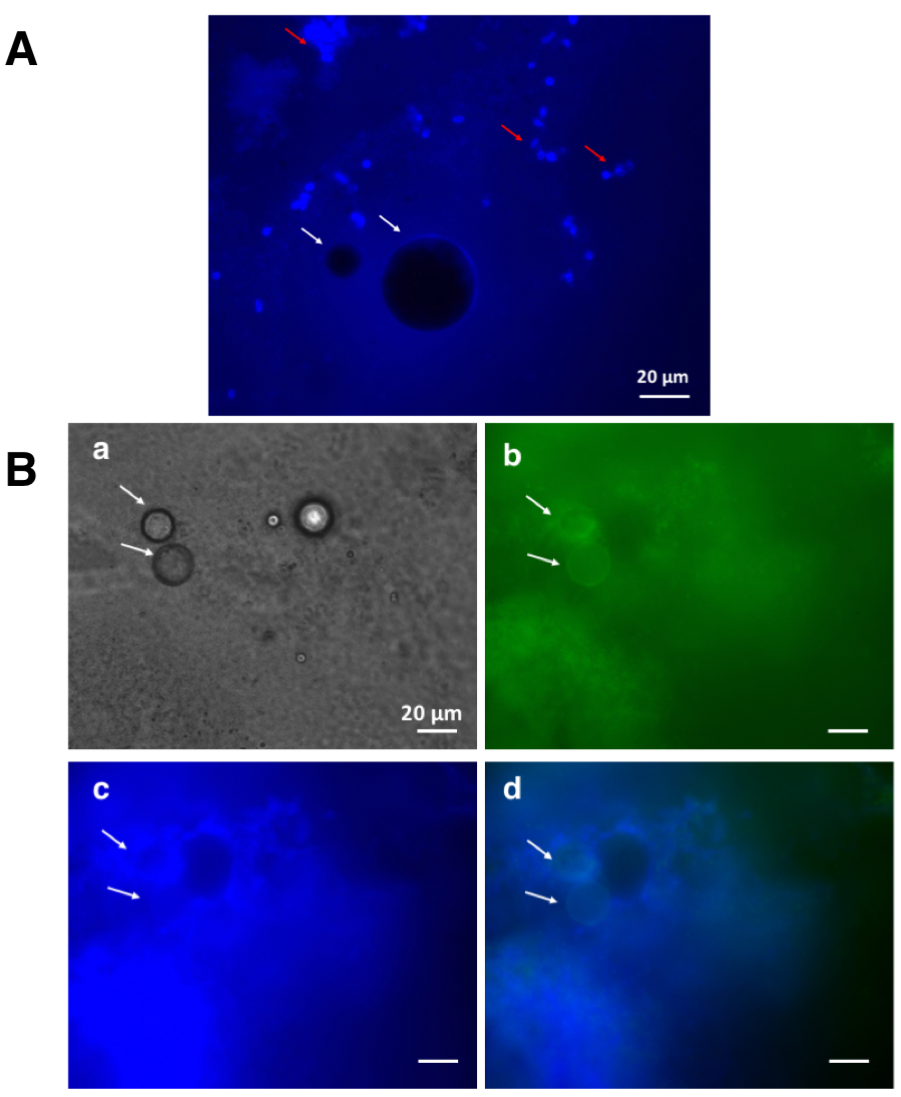


**Figure S4. A)** Representative fluorescence microscope images of cellulose attached to *n*-decane droplets. The blue colour surrounding the *n*-decane droplets (arrows in white) demonstrates the presence of cellulose. The red arrows indicate yeast cells. The scale bars denote 20 µm. **B)** Representative a) bright field, b) green fluorescence staining with SYTO 9, c) blue fluorescence staining with Calcofluor White, and d) superimposed green and blue fluorescence images of *Komagataeibacter* and cellulose adhering to *n*-decane droplets. The scale bars denote 20 µm. The emulsions of *n*-decane in microbial dispersions obtained through mixing kombucha suspension with *n*-decane were stained using SYTO 9 green fluorescent nucleic acid stain and Calcofluor White blue fluorescent stain that binds to cellulose and chitin. Chitin is found in the cell wall of yeasts, yielding the bright blue fluorescence of the yeasts surrounded by cellulose.

**
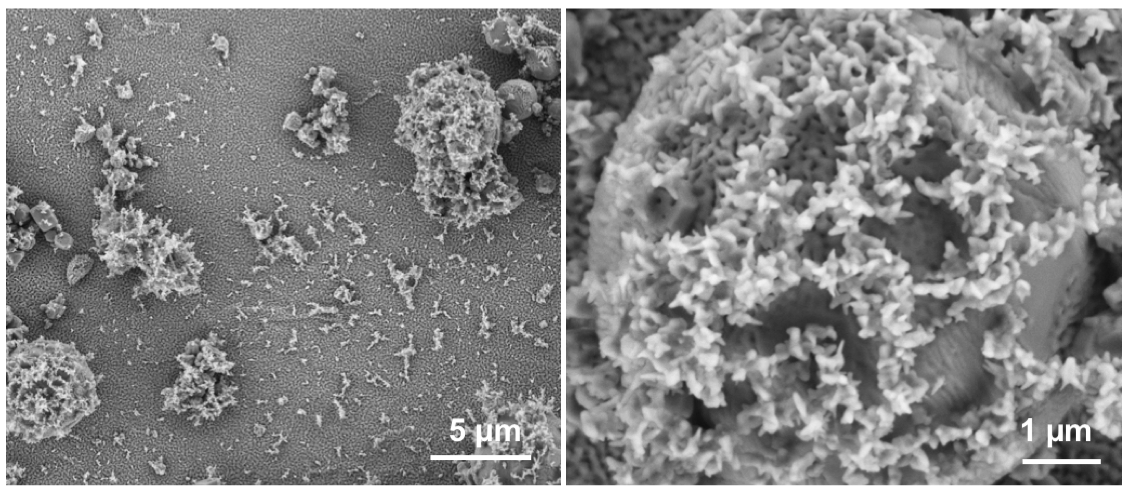
**

**Figure S5.** Cryo-scanning electron micrographs show protoplasts of yeasts with thick glucan fibrils.


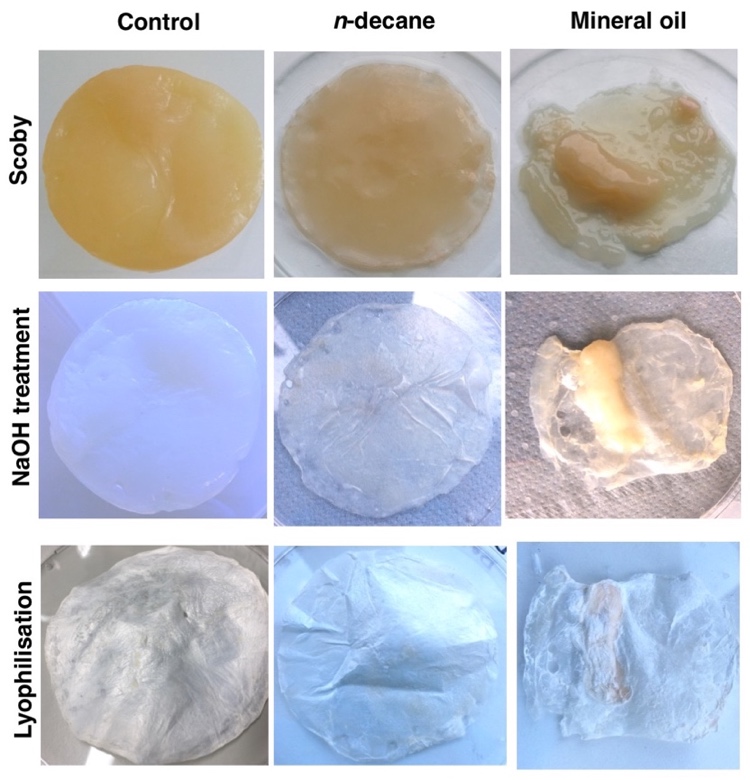


**Figure S6.** Cellulose membranes produced by *Komagataeibacter* in kombucha at the air-kombucha suspension (control) and oil-kombucha suspension interfaces. Representative photographs of kombucha pellicle formed at the interface after 30 days, after NaOH treatment to remove cellular material, and after lyophilization, respectively.


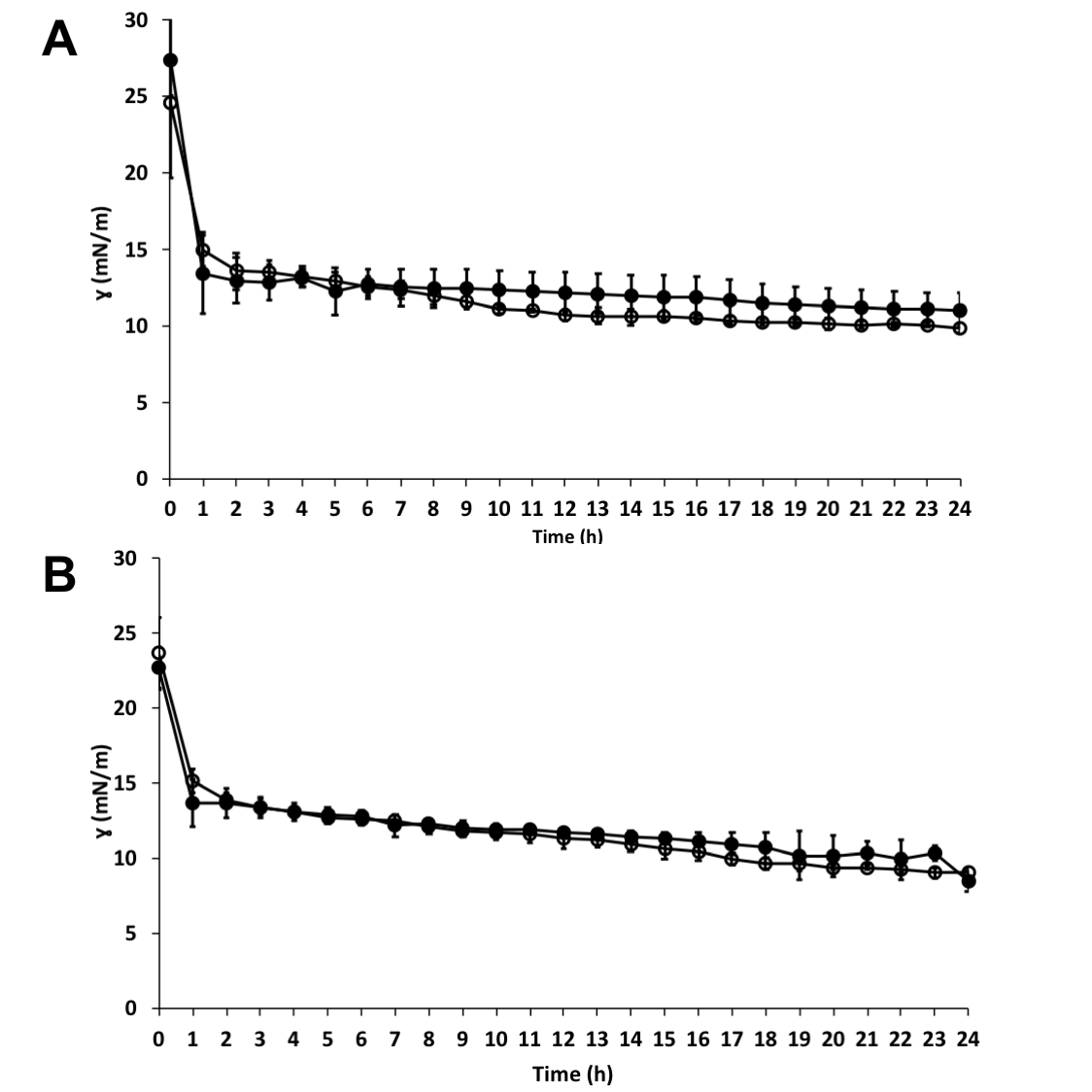


**Figure S7**. Interfacial tension ($\gamma$) as a function of time measured at the interface between **A**) *n*-decane and kombucha suspension, and **B**) mineral oil and kombucha suspension in supernatant (closed circle) and mineral oil and only supernatant without cells (open circle). The kombucha microbes-free suspension was prepared by centrifugation of cells at 5000 RPM for 5 min. After centrifugation, the supernatant was filtered through a 0.22 µm filter. Error bars represent standard deviations over three replicates.


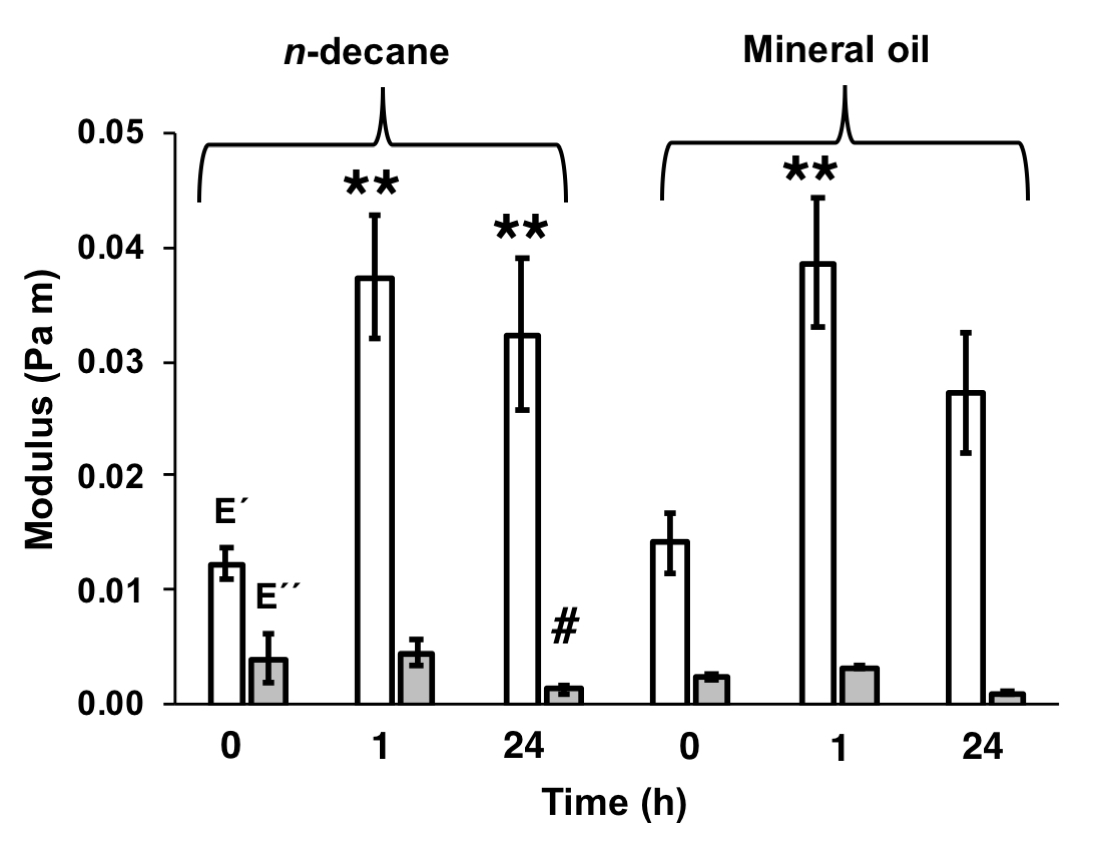


**Figure S8.** Rheology of bacterial adsorption and cellulosic biofilm formation at the oil-water interfaces. Interfacial elasticity measurements (E´- elastic modulus and E´´- viscous modulus) of bacterial adsorption and cellulosic biofilm formation at the *n*-decane/mineral oil-kombucha suspension interfaces performed by the oscillating drop method. The E´ and E´´ were measured at 0 h, 1 h, and 24 h. Error bars represent standard deviations over three replicates. ANOVA tests were performed, followed by a Tukey´s HSD post-hoc test, and a *p*-value <0.05 was considered significant. ** denotes significance (*p*<0.01) compared to 0 h. # denotes significance (*p*<0.05) compared to 0 h and 1 h at the *n*-decane-kombucha suspension interface.


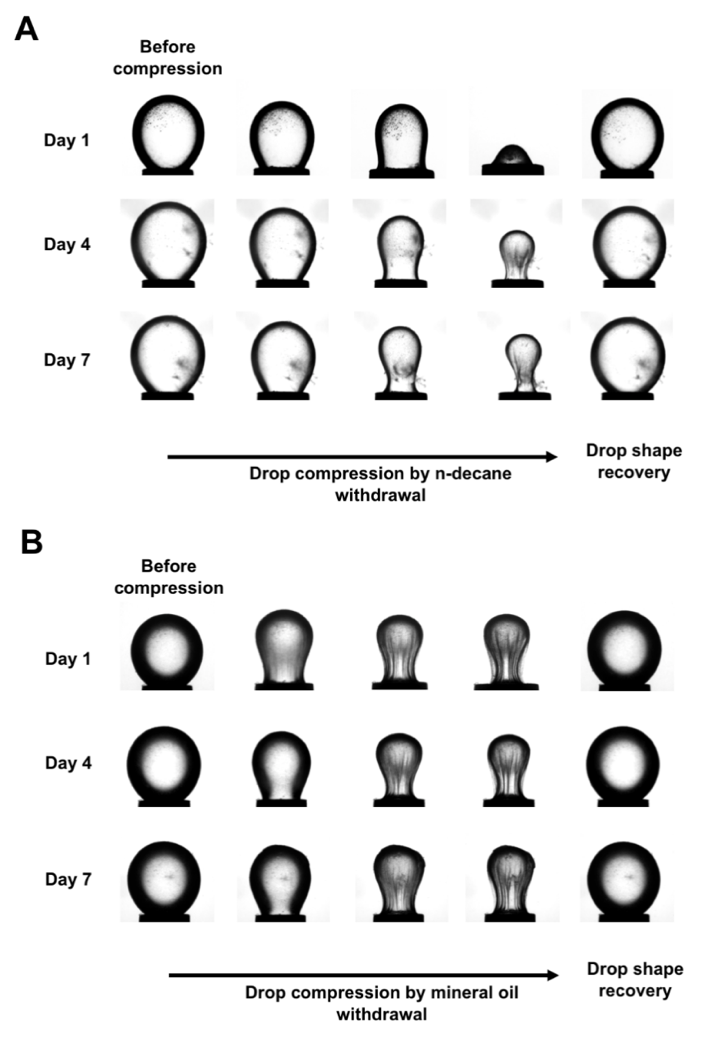


**Figure S9.** Deformation of interfaces of kombucha suspension in contact with **A**) *n*-decane or **B**) mineral oil droplet in response to a large volume reduction. A droplet of oil (5 µL) was formed at the tip of an inverted needle and aged in the bacterial suspension in PBS for 7 days. The aged drop is compressed manually every 24 h by withdrawing 90% of the oil droplet volume and then reinject it after observing the shape deformation of the biofilm at the droplet interface. The complete process (withdrawal and reinjection of oil droplet volume) took ~1 min.

**
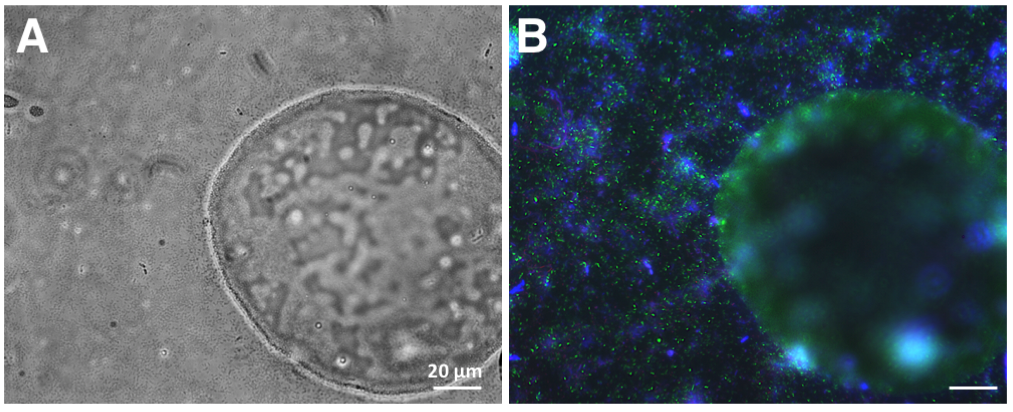
Figure S10.** Representative **A**) brightfield and **B**) fluorescence (superimposed of green, red and blue) images of cellulosic biofilms formed at mineral oil-kombucha suspension interface after 7 days. The scale bars denote 20 µm. The mineral oil drop formed at the tip of an inverted needle was aged in kombucha suspension for 7 days. The oil drop was then pipetted onto a clean glass slide, stained using vitality staining solution (3.34 mM SYTO 9 and 20 mM PI) and 25 µM Calcofluor White in PBS for 15 mins.

**Video S1**. A Stable cellulosic biofilm matrix formation at the air-kombucha suspension interface. An air bubble (5 µL) was formed at the tip of an inverted needle and aged in kombucha suspension for 13 h. When the air in the bubble was manually withdrawn, the cellulose matrix structure formed at the interface did not deform and remain stably attached to the tip of the inverted needle.

**Video S2**. Deformation of kombucha microbe interfaces resuspended in PBS in contact with an *n*-decane droplet in response to a large volume reduction. A droplet of *n*-decane (5 µL) was formed at the tip of an inverted needle and aged in kombucha suspension for 24 h. The aged drop is compressed manually by withdrawing the *n*-decane droplet volume. The volume was repeatedly withdrawn and reintroduced of *n*-decane.

**Video S3**. Deformation of kombucha microbe interfaces in PBS in contact with mineral oil droplet in response to a large volume reduction. A droplet of mineral oil (5 µL) was formed at the tip of an inverted needle and aged in kombucha suspension for 24 h. The aged drop is compressed manually by withdrawing the mineral oil droplet volume.
